# Chemical Constituents and Biologic Activities of Sage Species: A Comparison between *Salvia officinalis* L., *S. glutinosa* L. and *S. transsylvanica (Schur ex Griseb. & Schenk) Schur*

**DOI:** 10.3390/antiox9060480

**Published:** 2020-06-02

**Authors:** Andrei Mocan, Mihai Babotă, Anca Pop, Ionel Fizeșan, Alina Diuzheva, Marcello Locatelli, Simone Carradori, Cristina Campestre, Luigi Menghini, Cristian R. Sisea, Marina Sokovic, Gokhan Zengin, Ramona Păltinean, Sabin Bădărău, Dan C. Vodnar, Gianina Crișan

**Affiliations:** 1 Faculty of Pharmacy, “Iuliu Hațieganu” University of Medicine and Pharmacy, 8 Victor Babeș Street, 400012 Cluj-Napoca, Romania; mocan.andrei@umfcluj.ro (A.M.); mihai.babota@umfcluj.ro (M.B.); anca.pop@umfcluj.ro (A.P.); ionel.fizesan@umfcluj.ro (I.F.); rpaltinean@umfcluj.ro (R.P.); gcrisan@umfcluj.ro (G.C.); 2 Department of Pharmacy, “G. d’Annunzio” University of Chieti—Pescara, Via dei Vestini 31, 66100 Chieti, Italy; adyuzheva@gmail.com (A.D.); m.locatelli@unich.it (M.L.); simone.carradori@unich.it (S.C.); cristina.campestre@unich.it (C.C.); 3 Faculty of Horticulture, University of Agricultural Sciences and Veterinary Medicine, 400372 Cluj-Napoca, Romania; 4 Institute for Biological Research “Siniša Stanković”, University of Belgrade, Bulevar despota Stefana 142, 11060 Belgrade, Serbia; mris@ibiss.bg.ac.rs; 5 Faculty of Science, Selcuk University, Campus/Konya, 42250 Konya, Turkey; gokhanzengin@selcuk.edu.tr; 6 Faculty of Environmental Science and Engineering, Babeș-Bolyai University, 400084 Cluj-Napoca, Romania; alexandru@transsilvanica.net; 7 Faculty of Food Science and Technology, University of Agricultural Sciences and Veterinary Medicine, 400372 Cluj-Napoca, Romania; dan.vodnar@usamvcluj.ro

**Keywords:** *Salvia transsylvanica*, *S. glutinosa*, *S. officinalis*, bioactive compounds, phenolics, antioxidant compounds, enzyme inhibitory activity

## Abstract

Even though *Salvia* genus is one of the most known and studied taxa of Lamiaceae family, the knowledge regarding the chemical composition and health-related benefits of some locally used *Salvia* species (mostly endemic) is still scarce. In this regard, the present work aims to evaluate the chemical profile and potential bioactivities of 70% (*v/v*) ethanolic extracts obtained from the less-studied *S. transsylvanica* and *S. glutinosa* in comparison with *S. officinalis*. HPLC-PDA analysis revealed the presence of rutin and catechin as the main compounds in the extracts of the three studied species (using the employed HPLC method), whereas the presence of naringenin was highlighted only in *S. glutinosa* extract. Chlorogenic acid, rutin and quercetin were identified and quantified for the first time in *S. transsylvanica* extracts. The in vitro antioxidant capacity of each extract was tested through complementary methods (phosphomolybdenum assay, DPPH, ABTS, CUPRAC and FRAP assays), and correlated with the presence of phenolics (especially flavonoids) in high amounts. The neuroprotective and antidiabetic abilities of *S. officinalis* (the most active as AChE, BChE and α-glucosidase inhibitor), *S. glutinosa* (the most active as α-amylase inhibitor) and *S. transsylvanica* were also studied. For each extract it was determined the antimicrobial, antifungal and cytotoxic effects using in vitro assays. The obtained results confirm the potential of *S. transsylvanica* and *S. glutinosa* as promising sources of bioactive compounds and as a starting point for further analyses.

## 1. Introduction

The relationship between phytochemicals produced by plant metabolism and the effects revealed by empirical and scientific pharmacological observations fascinated researchers involved in the study of plant effects on human or animal health for centuries. Across the continents, cultures and centuries an incredible number of plants have been used for their beneficial effects. However, it is impossible to state why, when and who started their use. *Salvia* is a large genus of the Lamiaceae family that comprises over 1000 species distributed worldwide, with the virtual exception of Australia and New Zealand [1,2,3]. The genus *Salvia* could be considered highly dynamic for a large variety of medicinal, aromatic, cosmetic and ornamental applications, both as traditional and innovative uses. Also for taxonomists, the genus *Salvia* is still the object of investigations and recently was confirmed as non-monophyletic and re-circumscribed with relevant inclusion of other genera, such as *Dorystaechas*, *Meriandra*, *Perovskiam*, *Rosmarinus* and *Zhumeria* as *Salvia* species [4].

As one of the most known *Salvia* species, sage—particularly *Salvia officinalis*—can be considered a relevant example of a plant with an evidence-based use in traditional medicine as well in modern phytotherapy. It is officially recognized as a medicinal plant, with the leaves being used not only to prepare extracts for the treatment of skin disorders, minor wounds, mouth and throat disorders, but also in the treatment of gastrointestinal disorders in form of comminuted herb or aqueous and hydro-alcoholic extracts for oral use [5]. Chemical composition and relative variability of *S. officinalis* has been extensively investigated, and a plethora of factors have been recognized as relevant for qualitative and quantitative composition, such as environmental, physiological and morphologic factors, genotypes, age, environmental stress and agronomic procedures, climatic conditions, season, salinity and culture site [6,7]. The multipurpose applications of sage essential oil, derived from traditional use or as potential new approaches, including medicinal use, are largely supported by experimental phytochemical, biochemical and pharmacological studies as well as scientific reviews [8,9,10]. Polar fractions contain polyphenols, such as rosmarinic acid ellagic acid, rutin, chlorogenic acid, quercetin and a lower amount of luteolin-7-glucoside, epicatechin, epigallocatechin gallate. Phenolic acids such as caffeic acid and 3-caffeoylquinic acid are also present. Aqueous and alcoholic extracts contain also several volatile components such as borneol, cineole, camphor and thujone [11,12].

Sage is still one of the most common and widely used plants with numerous applications as a culinary herb—mainly for the volatile aroma; as medicine—due to the presence of polar and nonpolar biologically active metabolites and as cosmetic ingredient—with traditional and innovative uses, for examples as anti-hyperhidrotic agent for deodorant formulations. Moreover, the less investigated *Salvia* species, such as *S. transsylvanica*, may represent a promising and unexplored field of research for innovative products with medicinal properties. To the best of our knowledge, only two scientific papers have focused on this species. The most recent represents a comparative study on water–methanol extract from different *Salvia* species evidencing a promising antioxidant activity for *S. transsylvanica* directly related to the phenolic composition [13]. Previously, a preliminary in vivo pharmacological screening of 70% ethanol extract obtained from aerial parts of *S. transsylvanica* showed an active analgesic, antipyretic, antiepileptic, anti-inflammatory, antiulcerogenic and mild sedative potential [14]. As well, *S. glutinosa* L. is used in industry and traditional medicine, but with a low-evidence regarding phytochemical and pharmacological properties. The plant was studied for the antioxidant and antimicrobial potential of the essential oil, but also for other secondary metabolites such as flavonoids, other phenolics and sesquiterpenes [15,16].

The present study aims to elucidate and compare the phytochemical composition, the antioxidant capacity, enzyme inhibition activity (mainly focusing on their inhibitory capacities towards enzymes playing a key role in the management of sugar metabolism, melanogenesis and neurodegenerative disorders, such as Alzheimer’s disease) and antibacterial/antifungal effects of *S. glutinosa* and *S. transsylvanica* hydroalcoholic extracts. The main goal of our research is to compare the chemical profile and pharmacological effects of the extracts obtained from these species with the worldwide recognized properties of medicinal *S. officinalis*, in order to support their use as a valuable source of bioactive compounds with potential health benefits.

## 2. Materials and Methods

### 2.1. Chemicals and Reagents

The HPLC grade solvents (ethyl acetate (≥99%), acetonitrile, methanol, acetic acid (≥99%)) and Folin–Ciocalteu reagent were purchased from Merck (Darmstadt, Germany). The standards used for both spectrophotometric and HPLC analyses were: gallic acid, catechin, chlorogenic acid (3-CGA), 4-hydroxybenzoic acid, vanillic acid, epicatechin, syringic acid, 3-hydroxybenzoic acid, 3-hydroxy-4-methoxybenzaldehyde, *p*-coumaric acid, rutin, sinapinic acid, *t*-ferulic acid, naringin, 2,3-dimethoxybenzoic acid, benzoic acid, *o*-coumaric acid, quercetin, harpagoside, *t*-cinnamic acid, naringenin and carvacrol (purity > 98%) purchased from Sigma-Aldrich (Milan, Italy). Ultra-pure water was obtained using a Millipore Milli-Q Plus water treatment system (Millipore Bedford Corp., Bedford, MA, USA).

ABTS (2,2′-azino-bis(3-ethylbenzothiazoline-6-sulfonic acid) diammonium salt) ≥98% purity, potassium peroxodisulfate (≥99% purity), DPPH (2,2-diphenyl-1-picrylhydrazyl), Trolox (6-hydroxy-2,5,7,8-tetramethyl-chromane-2-carboxylic acid; ≥97% purity) kojic acid, acarbose, galantamine, streptomycin and ketoconazole also used in antioxidant, enzyme inhibition and antibacterial tests and were purchased from Sigma Aldrich (Schnelldorf, Germany). For cell culture assays, Dulbecco’s modified Eagle medium (DMEM) and phosphate-buffered saline (PBS) were purchased from Gibco (Paisley, UK), Eagle’s Minimum Essential Medium (EMEM) from ATCC (Manassas, VA, USA); resazurin and fetal bovine serum (FBS) were from Sigma Aldrich (Steinheim, Germany).

### 2.2. Plant Material

Aerial parts from *S. transsylvanica* (Schur ex Griseb. & Schenk) Schur (Băgău, Alba county, Romania), *S. glutinosa* L. (Vâlcea county, Romania) and *S. officinalis* L. (cultivated, Alba county, Romania) were collected from May to August 2014. The authenticity of plant material was confirmed after the identification procedure accomplished by dr. Andrei Mocan and dr. Sabin Bădărău. Plant material collection was performed to obtain representative samples without damage to the wild population (in case of the non-cultivated species). Samples were dried at room temperature to a constant weight and properly stored until the extraction.

### 2.3. Extraction Procedure

Samples were ground to a fine powder and sonicated at room temperature for 30 min using 1.50 g of plant material and 30.00 mL of 70% (*v/v*) ethanol. The extracts were filtered, alcohol being evaporated under reduced pressure using a rotary evaporator. Final solid extracts were stored in a desiccator at room temperature in a dark place until further analyses. For phytochemical analysis, antioxidant assays and enzyme inhibition tests the dry extracts were redissolved in the same solvent of extraction (ethanol 70%) while for the antifungal/antibacterial/cytotoxic activity, the dry extracts were dissolved in small amounts of DMSO and diluted in the growth medium in order to have a stock solution at concentration 1 mg mL^−1^ containing DMSO < 5%.

### 2.4. HPLC-PDA Analysis of Phenolic Compounds

Qualitative and qualitative analysis of phenolic compounds was performed according to a previously reported method [17], using a chromatographic system consisted of HPLC Waters liquid chromatograph instrument (model 600 solvent pump, 2996 PDA; Waters Spa, Milford, MA, USA). Mobile phase was directly degassed online by using a Biotech 4CH DEGASI Compact (Onsala, Sweden). A C18 reversed-phase column (Prodigy ODS (3), 4.6 × 150 mm, 5 μm; Phenomenex, Torrance, CA, USA), thermostated at 30 °C (±1 °C) was used for the separation of twenty-two phenolic compounds. The elution mode was applied with a mobile phase solution A (3% solution of acetic acid in water) and B (3% solution of acetic acid in acetonitrile) in a ratio 93:7 (*v:v*). The total separation was completed in 1 h. Empower v.2 software (Waters Spa, Milford, MA, USA) was used for data collection and analysis.

### 2.5. Determination of TPC (Total Phenolic Content) and TFC (Total Flavonoid Content)

The TPC was determined using a previously described Folin–Ciocalteu method using gallic acid as reference standard [18]. The content of phenolics was expressed as gallic acid equivalents (GAE) per gram of dry lyophilized extract (mg GAE/g dry extract). The TFC was evaluated using a method previously described by Mocan et al. [19]. Rutin was used as a reference standard and the TFC was expressed as mg rutin equivalents (RE) per gram of dry extract (mg RE/g dry extract). Both methods were adapted for microplate reader, measurements being made using a SPECTROstar Nano Multi—Detection Microplate Reader with 96-well plates (BMG Labtech, Ortenberg, Germany).

### 2.6. Antioxidant Capacity Assays

The antioxidant capacity of *S. glutinosa*, *S. transsylvanica* and *S.* officinalis extracts was evaluated through five in vitro methods (phosphomolybdenum assay, DPPH, ABTS, CUPRAC and FRAP assays) using a SPECTROstar Nano Multi-Detection Microplate Reader with 96-well plates (BMG Labtech, Ortenberg, Germany).

The total antioxidant capacity of the samples was evaluated using the phosphomolybdenum assay according to the protocol previously described [20]. First, 0.30 mL of extracts (2 mg mL^−1^) were combined with 3 mL reagent solution (0.6-M sulfuric acid, 28-mM sodium phosphate and 4-mM ammonium molybdate). The tubes containing the reaction solution were incubated at 95 °C for 90 min. Then, the absorbance of the solution was measured at 695 nm against a blank. The antioxidant capacity of extracts was expressed as mg equivalents of ascorbic acid (AE) per g of dry extract (mg AE/g extract).

The capacity to scavenge the DPPH free radical was monitored according to the method described by Martins et al. and Rusu et al. [21,22]. First, 30 µL of sample solution were mixed with a 0.004% methanolic solution of DPPH for 30 min in the dark. The absorbance at 517 nm was measured against a solvent blank. Trolox was used as a reference standard and the results were expressed as Trolox equivalents per gram of dry extract (mg TE/g dry extract).

The radical scavenging activity of the extracts against ABTS was measured according to Mocan et al. [23]. A Trolox calibration curve was plotted as a function of the percentage of ABTS radical cation scavenging activity. The final results were expressed as mg of Trolox equivalents (TE) per gram of dry extract (mg TE/g dry extract).

For the CUPRAC assay, the protocol previously reported by Apak et al. [24] was used with some modifications. Each sample was mixed with CuCl_2_ solution, neocuproine and ammonium acetate at pH 7.0; the absorbance of the mixture was measured at 450 nm after 30 min incubation, the final results being expressed as mg of Trolox equivalents per gram of dry extract (mg TE/g dry extract).

In the FRAP assay, the reaction mixture (25 µL of sample and 175 µL FRAP reagent) was incubated for 30 min at room temperature (in the dark), the absorbance of the blue-colored Fe^2+^-TPTZ (2,4,6-tris(2-pyridyl)-*s*-triazine) complex being measured at 593 nm. The FRAP reagent was freshly prepared by mixing ten volumes of acetate buffer (300 mM, pH 3.6), one volume of TPTZ solution (10-mM TPTZ in 40-mM HCl) and one volume of FeCl_3_ solution (20-mM FeCl_3_·6H_2_O in 40-mM HCl). The final results were expressed as mg of Trolox equivalents per gram of dry extract (mg TE/g dry extract) [25].

### 2.7. In Vitro Evaluation of Enzyme Inhibitory Properties of the Extracts

Inhibitory potential of the extracts on some key enzymes involved in neurological, metabolic and skin-related diseases was tested using in vitro assays adapted to a microplate reader. Hence, acetylcholinesterase (AChE) and butyrylcholinesterase (BChE) inhibitory properties (related to neuroprotective potential of the extracts) were investigated using protocols described by Menghini et al. [26] and Les et al. [27] using Ellman’s method with slight changes. The sample solution (2 mg/mL, 50 μL) was mixed with Ellman’s reagent DTNB (5,5-dithio-bis(2-nitrobenzoic) acid, 125 μL) and acetylcholinesterase (AChE) or butyrylcholinesterase (BChE) in buffer (Tris-HCl, pH 8.0, 25 μL). Successively, this mixture was incubated for 15 min, at 25 °C. Then, the substrates (acetylthiocholine iodide for AChE and butyrylthiocholine chloride for BChE, 25 μL) were added to this mixture. Blank solution was prepared without the enzyme. Solutions were incubated for 10 min at 25 °C and then the absorbances were measured at 405 nm in a 96-well microplate reader. Galantamine was used as a positive inhibitor and the results were expressed as mg galantamine equivalents (GALAE) per g of dry extract (mg GALAE/g dry extract).

The antidiabetic properties of the three *Salvia* extracts were tested based on their in vitro inhibitory capacity on *α*-amylase and *α*-glucosidase [25,27]. In *α*-amylase assay, sample solutions (2 mg/mL, 25 μL) were premixed with the α-amylase solution (50 μL) in phosphate buffer (pH 6.9) After 10 min at 37 °C, a starch solution (50 μL, 0.05%) was added to these mixtures. The final mixtures were left to stand for 10 min at 37 °C and HCl solution was used to stop the reaction (1 mM, 25 μL). Blank solution was prepared without the enzyme. Finally, the iodine–potassium iodide reagent was added and the absorbances were read at 630 nm in a 96-well microplate reader, using acarbose as positive standard. For *α*-glucosidase assay, the extracts (2 mg/mL, 50 μL) were mixed with 50 μL of glutathione (2 mg/mL), 50 μL of enzyme (in phosphate buffer, pH 6.8) and 50 μL of substrate (PNPG, 10 mM). After 15 min at 37 °C, 50 μL of sodium carbonate were added to stop the reaction. The final absorbances were measured at 400 nm in a 96-well microplate reader using acarbose as positive standard. For both assays, the final results were expressed as mg acarbose equivalents (ACAE) per g of dry extract (mg ACAE/g dry extract).

Tyrosinase inhibitory activity of each sample was determined by a method previously described [25]. Samples were dissolved in water containing 5% DMSO; for each sample four wells were designated as A, B, C, D; each one contained a reaction mixture (200 µL) as follows: (A) 120 µL of 66 mM-phosphate buffer solution (pH = 6.8) (PBS), 40 µL of mushroom tyrosinase in PBS (46 U/mL) (Tyr), (B) 160 µL PBS, (C) 80 µL PBS, 40 µL Tyr, 40 µL sample and (D) 120 µL PBS, 40 µL sample. The plate was then incubated at room temperature for 10 min; after incubation, 40 µL of 2.5 mM-L-DOPA in PBS solution were added in each well and the mixtures were incubated again at room temperature for 20 min. The absorbance of each well was measured at 475 nm using kojic acid as positive control, final results being expressed as mg of kojic acid equivalents (KAE) per g of dry extract (mg KAE/g dry extract).

### 2.8. Antibacterial Properties of the Extracts

#### 2.8.1. Antibacterial Activity

Antibacterial activity of the extracts from the three sage samples was tested against a set of three Gram-positive bacteria, namely *Bacillus cereus* (clinical isolate), *Micrococcus flavus* (ATCC 10,240) and *Staphylococcus aureus* (ATCC 6538) and five Gram-negative strains, *Escherichia coli* (ATCC 35210), *Pseudomonas aeruginosa* (ATCC 27853), *Salmonella typhimurium* (ATCC 13311), *Listeria monocytogenes* (NCTC 7973), *Enterobacter cloacae* (clinical isolate). According to a previously described microdilution method [28] the antibacterial activity was tested on 96 wells microplates.

Briefly, a bacterial suspension 0.5 McFarland in sterile physiological solution was adjusted to obtain 1.0 × 10^5^ bacterial cells in a final volume of 100 μL per well. Each dry extract was solubilized in DMSO and diluted in tryptic soy broth (TSB) medium (100 μL) to a final concentration of stock solution 1 mg mL^−1^, containing DMSO lower than 5%. From the stock solution, a serial dilution (3.0–0.01 mg mL^−1^) of extracts in culture medium was prepared in 96 wells microplate. Two reference drugs were used as positive controls, namely streptomycin and ampicillin (1 mg mL^−1^ in sterile physiological saline). The medium was then inoculated (1.0 × 10^4^ CFU per well) and after 24 h at 37 °C, 30 μL of 0.2 mg mL^−1^ solution of INT (*p*-iodonitrotetrazolium violet) were added and placed in an incubator for 1 h in order to allow the change of the yellow dye to pink, due to the reductive metabolism of viable cell bacteria [29]. Clear or pale yellow solution indicates no bacterial growth, while purple solution indicates the presence of living bacteria. The presence/absence of microbial growth was determined by a binocular microscope and the lowest concentration with no bacterial growth was recorded as minimal inhibitory concentration (MICs). The minimal bactericidal concentrations (MBCs) were determined sub-culturing 2 μL of the medium containing the extract into 100 μL of medium broth per well. Then, 24 h later, the lowest concentration with no visible growth was defined as the MBC. Each test was performed in triplicate and at least three independent experiments were performed.

#### 2.8.2. Antiquorum and Antibiofilm Activity

*Bacterial strains, growth media and culture conditions.* A strain of *P. aeruginosa* PA01 (ATCC 27853) from the Mycoteca of Institute for Biologic Research “Sinisa Stankovic” (Belgrade, Serbia) was used to perform the test. Bacteria were incubated in Luria–Bertani (LB) medium (1% *w/v* NaCl, 1% *w/v* tryptone, 0.5% *w/v* yeast extract) at 37 °C under slight agitation.

*Biofilm formation.* Each extract was tested as inhibitory agent for biofilm formation on 96 well microplates. Extracts were tested at subinhibitory concentrations obtained by diluting 1/2, 1/4 and 1/8 the MIC concentration. The used experimental procedure is derived from Drenkard et al. [30], with slight modifications, as previously reported [31]. Briefly, from the 24-h culture of *P. aeruginosa* (inoculum concentration 1 × 10^8^ CFU mL^−1^) an aliquot of 100 μL per well was mixed with an equal volume of medium supplemented with subinhibitory concentrations (subMIC) of extracts (0.5, 0.25 and 0.125 MIC) and incubated for 24 h at 37 °C. As a negative control, basal medium was used, while for positive control, medium containing same dilution of ampicillin and streptomycin were used. After 24 h the medium was removed, adherent cells were washed with sterile PBS (pH 7.4) and air dried. The biofilm mass was defined by staining for 10 min with 0.1% solution of crystal violet. The excess stain was removed by gentle washing with dH_2_O and 200 μL of 95% ethanol (*v/v*) was added to solubilize the dye that had stained the biofilm cells. The absorbance of the ethanol solution was recorded at λ = 625 nm on a Sunrise™ Tecan ELISA reader (Mannedorf, Switzerland). Results are expressed as percentage inhibition of biofilm formation compared to the control untreated group. The experiment was performed in triplicate and repeated twice, and values were presented as mean values ± SE.

*Inhibition of P. aeruginosa biofilm formation. P. aeruginosa* was cultured overnight at 37 °C in LB medium and adjusted to a concentration of 1.0 × 10^8^ CFU mL^−1^ for final inoculum. Solutions at scalar subMIC concentrations of extracts (range 0.04 to 0.1 mg mL^−1^ for *S. officinalis* and 0.75-0.18 mg mL^−1^, for *S. trannsylvanica* and *S. glutinosa*, respectively) were adsorbed on sterile filter study discs (Whatman; 4 mm in diameter, streptomycin and ampicillin 0.125, 0.25, 0.5 mg per disc). Discs were taken to dryness and placed on Petri dish inoculated with *P. aeruginosa* (1.0 × 10^8^ CFU mL^−1^) and incubated at 37 °C. After 24 h of incubation, effects of growth inhibition or antiquorum zones were recorded. The presence of a clear zone around the discs with no bacterial colony indicates an effect of growth inhibition measured as the diameter of inhibition. The antiquorum effects, i.e., antibiofilm zones were detected as different growth characters of the colony margin in proximity of inhibition zone.

*Inhibition of synthesis of P. aeruginosa PA01 pyocyanin*. Fresh liquid culture of *P. aeruginosa* PA01 was adjusted to 0.2 measured at OD600 nm and aliquots of 4.75 mL were distributed in culture tubes. Then 250 μL of a sterile solution containing plant extracts or reference drugs were added to obtain a final concentration of 0.5 MIC (0.045 mg mL^−1^ for *S. officinalis*, 0.75 mg mL^−1^ for *S. transsylvanica* and *S. glutinosa*, 0.2 mg mL^−1^ for ampicillin and 0.065 mg mL^−1^ streptomycin). Then, 250 μL of sterile saline solution were added to control untreated group. After 24 h of incubation, a liquid–liquid extraction of the medium with chloroform (3 mL) was performed and the chloroform phase acidified with 0.2-M HCl (1 mL) giving a pink to deep red solution. The OD of acidified organic phase was recorded at 520 nm using a Shimadzu UV1601 spectrophotometer (Kyoto, Japan). Results are expressed as percentage variation of pyocyanin production compared to untreated control *P. aeruginosa* PA01. The experiment was done in triplicate and repeated two times and results expressed as mean values ± SE.

#### 2.8.3. Antifungal Activity

In order to evaluate the effect of extracts supplementation on fungal growth, a pool of strains relevant for safety in the agri-food chain due to common presence in stored food and production of a large number of mycotoxins was used. The used tested strains were: *Aspergillus fumigatus* (plant isolate), *Aspergillus versicolor* (ATCC 11730), *Aspergillus ochraceus* (ATCC 12066), *Aspergillus niger* (ATCC 6275), *Trichoderma viride* (IAM 5061), *Penicillium funiculosum* (ATCC 36839), *Penicillium ochrochlorum* (ATCC 9112), *Penicillium verrucosum* (food isolate). The antifungal activity was investigated using the microdilution method, as already described [31].

Briefly, a fungal spore suspension was obtained by gentle washing with 0.85% saline containing 0.1% Tween 80 (*w/v*) the surface of strain growing on Petri dish. The solution was diluted in Sabouraud broth medium (SDA) and an aliquot of 100 μL containing 1.0 × 10^5^ spores was placed in each well. To each well, an equal amount (100 μL) of medium (SDA) in the presence/absence of scalar concentration (3.0–0.01 mg mL^−1^) of plant extracts or reference antifungal agents was added. Dilutions of extracts were prepared from stock solutions obtained from dry extracts solubilized (5 mg mL^−1^) in DMSO that results in a final concentration lower than 5%. Ketoconazole and bifonazole (0.10–3.50 mg mL^−1^) were used as positive controls, whereas no extract supplementation was used as control untreated group. After 24 h at 28 °C of incubation on a rotary shaker (160 rpm) the fungal growth was visually detected using a binocular microscope and the lowest concentration with no hyphal growth was defined as MICs. To define the minimum fungicidal concentrations (MFCs) a serial subcultivation of 2-μL aliquots was placed into microtiter plates containing 100 μL of fresh liquid medium. After incubation for 72 h at 28 °C, the presence of visible growth was detected and the lowest concentration with no growth was defined as MFC indicating 99.5% killing of the original inoculums. Experiments were performed in triplicate.

### 2.9. Biologic Activities of Salvia Extracts on Cell Lines

#### 2.9.1. Cell Culture

Human lung adenocarcinoma (A549), human breast cancer (MCF-7) and human hepatocellular carcinoma (HepG2) were obtained from American Type Culture Collection (ATCC, Manassas, VA, USA), while human normal gingival fibroblasts (HGF) were obtained from CLS Cell Lines Service (Eppelheim, Germany). A549, MCF-7 and HGF were maintained in Dulbecco’s Modified Eagle Medium (DMEM), while HepG2 were maintained in Eagle’s Minimum Essential Medium (EMEM), both media being supplemented with 10% fetal bovine serum (FBS). Cells were cultured at 37 °C in a humidified incubator with 5% CO_2_ supplementation, the medium was changed every 2–3 days and the cells were subcultured once they reached 70–80% confluence.

#### 2.9.2. Preparation of Extract Solutions and Cytotoxicity Assay

A 50 mg/mL stock solution was prepared in dimethyl sulfoxide (DMSO) for each *Salvia* extract. The stock solution was further diluted in DMSO to obtain working solutions of 0.39, 0.78, 1.56, 3.125, 6.25, 12.5 and 25 mg/mL. These working solutions were then used to obtain the desired concentrations in the cell culture medium. Harvested cells were seeded in 96 well plates and left to attach to the plastic substrate. After 24 h, the cells were washed with PBS and further exposed for 24 h/48 h to the three *Salvia* extracts. Following the exposure and a subsequent wash with PBS, the cells were exposed to a resazurin solution of 200 µM for 2 h and the fluorescence was measured at λ_excitation_ = 530/25; λ_emission_ = 590/35, using Synergy 2 Multi-Mode Microplate Reader.

The experiment was conducted using three biologic replicates, each one including 6 technical replicates. The obtained results were calculated as relative values compared to the negative control (cells exposed to vehicle; 0.2% DMSO in cell culture medium). To facilitate the interpretation of the results, when possible, IC_50_ values were calculated from the dose–effect curves obtained by fitting the experimental data with a 4-parameter logistic curve in SigmaPlot 11 software.

## 3. Results and Discussions

Phenolics are described as a main class of bioactive compounds found in *Salvia* species, being represented both in polar and nonpolar fractions of the extracts obtained from these plants [13,14]. Among these compounds, caffeic acid and its derivatives, like rosmarinic acid, were cited by the literature as the most abundant in the ethanolic extracts of different *Salvia* species (i.e., *S. officinalis, S. recognita, S. scabiosifolia, S transsylvanica*) [13]. Hence, our screening was focused on the qualitative and quantitative phenolic profile of the extracts obtained from *S. transsylvanica, S.* glutinosa and *S. officinalis*; 22 standard compounds were used, but only 10 of them were identified and quantified in the analyzed extracts (Table 1).

For all three species, rutin (the highest amount found in *S. glutinosa* extract—4070.2 ± 636.5 µg/g) and catechin (the highest amount found in *S. transsylvanica* extract—1911.1 ± 11.2 µg/g) were found as main compounds. Epicatechin was found only in *S. transsylvanica* (569.2 ± 25 µg/g) and *S. officinalis* (1659.1 ± 10.8 µg/g) extracts, while naringenin could be quantified only in *S. glutinosa* extract (828.5 ± 253.9 µg/g). The presence of carvacrol (usually found in volatile fractions of aromatic plants) was also highlighted in all extracts. Chlorogenic acid (3-CGA) was quantified only in *S. transsylvanica* and *S. glutinosa* extracts, even though its presence was previously reported by other authors for *S. officinalis* [9]. This could be explained based on the multiple isomeric forms of this compound, such as 5-CGA and 3-CGA, frequently leading to confusions due to their similarity [32].

One of the main goals of the present research was to evaluate the phenolic profile of less studied *S. transsylvanica* and *S. glutinosa*. Regarding the first species, Janicsák et al. [13] also reported the presence of rosmarinic acid (3.09% in dry leaves) and caffeic acid (0.11% in dry leaves) after extraction with methanol. To the best of our knowledge, the current study is the most comprehensive study on the phenolic profile of *S. transsylvanica*, bringing new data about the presence of some polyphenolic compounds with health-related benefits (such as chlorogenic acid, rutin, quercetin) beyond those yet studies by Janicsák et al. [13]. *S. glutinosa* was previously studied for the presence of different phenolic compounds, especially flavonoids. Veličković et al. [33] studied the chemical composition of the extracts obtained by classical maceration and ultrasonication from *S. glutinosa* aerial parts, revealing the presence of flavonoidic compounds (kaempferol, apigenin and quercetin derivatives) and caffeic acid. Our results confirm for the first time the presence of *p*-coumaric acid (998.9 ± 239.5 µg/g), naringenin (668.9 ± 55.9 µg/g) and rutin (4070.2 ± 636.5 µg/g) in *S. glutinosa* aerial parts extract, emphasizing the importance of this species as an alternative source of rutin. Also known as rutoside or quercetin 3-rutinoside, rutin was intensively studied for its potential bioactivities, being recommended for the treatment of various diseases such as varicose veins, internal bleeding or hemorrhoids, as well as dietary flavonoid with antimicrobial, anti-inflammatory and antidiabetic potential [34].

### 3.1. Determination of TPC (Total Phenolic Content) and TFC (Total Flavonoid Content)

Several studies revealed the importance of phenolics, especially flavonoidic compounds, as secondary metabolites responsible for multiple bioactivities of *Salvia* species. In this line, we aimed to evaluate total phenolic and total flavonoidic content of *S. transsylvanica, S. glutinosa* and *S. officinalis* aerial parts extracts, the results being presented in Table 2.

The highest values of TPC and TFC were found for *S. officinalis* extracts (65.02 ± 2.44 mg GAE/g extract, respectively 31.80 ± 0.24 mg RE/g extract), followed by *S. glutinosa* (37.74 ± 0.69 mg GAE/g extract, respectively 16.03 ± 0.40 mg RE/g extract). The lowest concentrations were determined in *S. transsylvanica* extracts (TPC—20.32 ± 1.01 mg GAE/g extract, TFC—13.94 ± 0.55 mg RE/g extract). A comparative study made by Janicsák et al. [13] evaluated the total phenolic content for 11 *Salvia* species, including cultivated *S. officinalis* and *S. transsylvanica*. The results showed that *S. transsylvanica* contains 8.4% phenolics (expressed in g of caffeic acid per 100 g of dry leaves), more than *S. officinalis* (6.44%). The different results reported by Janicsák et al. can be explained by the different way of expressing the results, but also by the individual variables of plant material (i.e., provenience, growing and harvesting conditions). In the above-mentioned study, the plant material derived from a cultivation crop, while in our study the plant was harvested from spontaneous flora, emphasizing that cultivated *S. transsylvanica* could produce higher amounts of phenolics than the exemplars from spontaneous flora. In a previous study, Veličković et al. [16] analyzed the TPC and TFC of 70% (*v/v*) ethanolic and methanolic extracts obtained from *S. glutinosa* and *S. officinalis* aerial parts using maceration and ultrasound-assisted extraction. For *S. glutinosa*, the results varied from 66.9 ± 0.50 to 137.3 ± 0.42 mg gallic acid/g of dry extract for TPC and from 60.3 ± 0.78 to 108.5 ± 0.57 mg rutin/g of dry extract (depending on extraction solvent and method used). The highest values were obtained using ultrasound-assisted extraction and 70% ethanol (*v/v*) as solvent, a similar trend being observed for *S. officinalis* extracts. Hence, our results are in line with previously reported studies on *S. glutinosa* and *S. officinalis* and argue the potential use of *S. glutinosa* as an alternative source of phenolics with bioactive properties.

### 3.2. Antioxidant Capacity Assays

The antioxidant potential of the extracts obtained from *S. transsylvanica, S. glutinosa* and *S. officinalis* was evaluated using five complementary assays (Table 3).

The obtained results varied from each test, but, except metal chelating assay, we can notice that for each sample, antioxidant capacity is correlated with the content in phenolic/flavonoidic compounds. This trend can be explained based on the general hypothesis that the plants rich in polyphenols and flavonoids exert an important antioxidant activity. For sure, the antioxidant capacity of the extracts can be also correlated with the presence of other compounds (i.e., terpenoids—carnosic acid, carnosol, iridoids) with a proven antioxidant potential [35]. Regarding the studied species, the best antioxidant activity was proven for *S. officinalis* extract, both in CUPRAC and ABTS assays (400.01 ± 4.41 mgTE/g extract and 358.56 ± 6.37 mgTE/g extract), followed by *S. glutinosa* (175.91 ± 6.18 mgTE/g extract in CUPRAC assay, respectively 130.59 ± 4.71 mgTE/g extract in FRAP assay). *S. transsylvanica* exerted the lowest antioxidant capacity in all assays. Concerning the metal chelating assay, according to Rice-Evans et al. metal chelating plays a minor role in the overall antioxidant activities of some phenolic compounds/phenolic enriched extracts [36] and the antioxidant responses of certain samples can be ascribed as well to non-phenolic chelators.

Similar results on *S. transsylvanica* were reported by Maklad et al. [14] who measured the antioxidant capacity of 11 *Salvia* species (including *S. transsylvanica*) via inhibition of the autooxidation of unsaturated fatty acids present in rat brain tissue. Even though the samples of *S. transsylvanica* were cultivated (and in our study collected from wild flora), the results revealed a medium-low antioxidant potential for the extracts (EC_50_ 6.5–7.0 µg/mL). These results encourage future investigations regarding the correlation between the phytochemical profile of this species and their antioxidant capacity, to establish the main compounds responsible for the antioxidant potential.

### 3.3. In Vitro Evaluation of Enzyme Inhibitory Properties of the Extracts

The extracts were evaluated for their potential benefits as neuroprotective, antidiabetic and skin-related diseases agents using enzyme inhibition assays, the results being summarized in Table 4.

Acetylcholinesterase (AChE) and butyrylcholinesterase (BChE) inhibitory activity of the extracts was tested in order to screen the potential of studied *Salvia* species as neuroprotective agents. *S. officinalis* exerted the most important inhibitory activity on both enzymes, the best results being obtained for BChE (2.40 ± 0.16 mgGALAE/g extract). Several studies were focused on the evaluation of neuroprotective properties of *S. officinalis* (including clinical trials) [37,38], the AchE and BchE inhibitory activity being correlated with the presence of high amounts of rosmarinic acid, carnosic acid and quercetin in polar fractions of the extracts [37].

For *S. transsylvanica* and *S. glutinosa* AchE inhibitory potential was comparable (1.72 ± 0.09 mg GALAE/g extract and 1.70 ± 0.16 mg GALAE/g extract), while the anti-BchE activity was almost three-fold higher for *S. transsylvanica* (1.43 ± 0.19 mgGALAE/g extract) than for *S. glutinosa* (0.52 ± 0.10 mgGALAE/g extract). The obtained results could be linked to the presence in high amounts of some phenolics (i.e., catechin, rutin) and monoterpenoidic (i.e., carvacrol) compounds (previously reported for their BChE inhibitory potential [34,39,40]) in the extract of *S. transsylvanica*.

Antidiabetic potential of the extracts was evaluated through their *α*-amylase and *α*-glucosidase inhibitory activity, both enzymes playing a key role in sugars metabolic pathways. All species exerted a higher inhibitory activity on *α*-glucosidase, the most active being *S. officinalis* (27.01 ± 0.12 mmol ACAE/g extract), followed by *S. transsylvanica* (25.62 ± 1.10 mmol ACAE/g extract) and *S. glutinosa* (21.54 ± 1.29 mmol ACAE/g extract). Conversely, the values obtained for *α*-amylase assay were small. Our results are in line with previously reported in vivo study conducted by Alarcon-Aguilar et al. [41]. The hypoglycemic effects of hydroethanolic extracts of *S. officinalis* were tested on healthy and alloxan-induced diabetic mice, revealing hypoglycemic activities in normoglycemic mice and in mildly diabetic mice induced by alloxan; these effects were correlated with the presence of polar hypoglycemic components in the extract, but no supplementary phytochemical investigations were made. Finally, the Salvia extracts did not reveal any inhibitory activity of tyrosinase.

To the best of our knowledge, our study reports for the first-time new data about AChE, BChE, *α*-amylase and *α*-glucosidase inhibitory properties of the extracts obtained from *S. transsylvanica* and *S. glutinosa*. The present study opens new perspectives for future in vitro and in vivo investigations that could elucidate the intimate mechanisms of action of this species as natural sources for neuroprotective and hypoglycemic agents.

### 3.4. Antimicrobial Properties of the Extracts

#### 3.4.1. Antibacterial Activity

Data on antimicrobial activity (Table 5) revealed that all extracts were active against bacteria, but with different efficacy. From the tested extracts, *S. officinalis* extract was the most efficient antibacterial agent with a broad spectrum of action, displaying MIC values lower than 1 mg mL^−1^ against all tested strains. In case of *S. transsylvanica* and *S. glutinosa* the MIC values ranged between 1.5–0.75 and 1.5–0.09 mg mL^−1^, respectively.

Only *B. cereus* was more sensitive to *S. glutinosa* extract (MIC value of 0.09 mg mL^−1^) while *S. officinalis* was more efficient against all other strains. The extract of *S. transsylvanica* showed a MIC value lower than *S. glutinosa* against *S. aureus*, while the latter was more active against *E. coli*, *E. cloacae* and *B. cereus*. The most sensible strains were the coliform *E. cloacae* (MIC 0.01 mg mL^−1^) followed by *E. coli* (MIC 0.045 mg mL^−1^), *S. typhimurium* and *P. aeruginosa* to *S. officinalis* extract, while the *B. cereus* strain was sensible to *S. glutinosa* extract (MIC 0.09 mg mL^−1^). Furthermore, the activity of *S. officinalis* extract against the cited strains was stronger than those expressed by the reference drugs.

The selective activity of *S. officinalis* extract on *E. cloacae* was also confirmed in experiments of Bouajaj et al. [42] who reported the above-mentioned strain to be highly sensitive to sage essential oil. Conversely, in the study of Mekinić et al. [43] an 80% ethanolic extract of *Salvia* showed higher activity on the Gram-positive *B. cereus* and *S. aureus*, (MIC 1.68 and 0.34 mg mL^−1^, respectively) compared to Gram-negative bacteria such as *E. coli* (MIC 6.78 mg mL^−1^) [43]. The different antibacterial activity could be mainly related to the phytochemical profile that registered relevant qualitative (in our samples are present metabolites as epicatechin, *o*-coumaric acid and *t*-cinnamic acid while quercetin, *t*-ferulic acid that are reported in the study of Mekinić et al. [43] were not detected) as well as quantitative differences.

*E. cloacae* is a part of the intestinal and skin microflora, but can become pathological when it infects different apparatus, such as respiratory or urinary tracts, conditions that are not rare in immunosuppressed subjects or hospitalized patients. The pharmacological treatment of *E. cloacae* infection is not easy due to the increasing resistance to classical antibiotics, such as penicillins, cephalosporins and amoxicillin/clavulanic acid [44]. The selective antibacterial effect of the two sages against this strain suggests a potential protective and preventive effects derived from regular food use of *S. officinalis* and *S. glutinosa*, as well as from innovative medical herbal preparations or cosmetic formulation, such as intimate wash. In the current study, the strong antibacterial activity of *S. officinalis* was oriented against the Gram-negative bacteria in accordance with Veličković et al. [16], while no specific efficiency was observed for *S. glutinosa* that registered the strongest inhibition on the Gram-positive *B. cereus* and the Gram-negative *E. cloacae*. Moreover, for the less active *S. transsylvanica* extract, no selectivity of action towards the Gram-negative or Gram-positive strains was observed. The sensitivity scale to *S. officinalis* and *S. glutinosa* extracts for selected strains were *E. coli* > *P. aeruginosa* > *S. aureus*, being in concordance to experimental data of disk diffusion test reported for 70% ethanol and methanol extracts [16].

#### 3.4.2. Antiquorum and Antibiofilm Activity

The prominent antibacterial activity of *S. officinalis* was confirmed by *P. aeruginosa* biofilm inhibition test (data are reported in Table 6), the highest tested concentration (0.5 MIC, 0.045 mg mL^−1^) of *S. officinalis* extract showing the strongest biofilm inhibition (over 60%). Interestingly, also the extracts of less active sage species had a relevant antibiofilm activity. The results for *S. transsylvanica* and *S. glutinosa* were comparable to ampicillin, with an inhibition percentage of 48% for *S. transsylvanica* and 30% for *S. glutinosa*, respectively; as well, at lower concentration (0.25 MIC, corresponding to 0.022 mg mL^−1^ and 0.375 mg mL^−1^, for *S. officinalis* and *S. transsylvanica*, respectively).

Noteworthy, at the lowest tested concentration (0.125 MIC) all sage extracts revealed marginal biofilm inhibition (10–19%), inhibition that was slightly higher than that of the reference drugs. The latter evidence suggests new potential interest in the applicative use of sage extracts as microbial control agents with a mechanism of action that is not directly related to the bacteriostatic/bactericidal activity.

The above-mentioned hypothesis is corroborated with the results regarding the inhibition of pyocyanin production. Pyocyanin production is a recognized marker of bacterial virulence, both in vitro and in vivo conditions. As reported in Table 7, only the treatment with 0.5 MIC of *S. transsylvanica* extract determined a reduction of pyocyanin (–28%), reduction that was slightly stronger than that exerted by the reference drugs. These results, coupled with the weak antibacterial activity of *S. transsylvanica*, suggests an unknown, but peculiar, mechanism of interaction with bacterial metabolism.

#### 3.4.3. Antifungal Activity

The effects of scalar dilutions of extracts added to fungal growth medium are reported as MIC and MFC in Table 8.

The fungal tested strains were sensitive to all *Salvia* extracts, but a wide range of MIC was observed (min 0.06 mg mL^−1^ on *P. verrucosum* and max 3 mg mL^−1^ on *P. ochrochlorum*). *S. officinalis* was the most effective antifungal agent with MIC values lower than other extracts and in some cases, comparable to reference drugs. Furthermore, *S. officinalis* had a broad spectrum of action, with all tested strains being affected by the presence of the extract in the medium. The most sensitive strains to *S. officinalis* were *P. funiculosum* (MIC 0.06 mg mL^−1^) and *P. verrucosum* (MIC 0.12 mg mL^−1^). Considering a cutoff at 1 mg mL^−1^ as an indicator for a relevant antifungal activity, only *P. ochrochlorum* was not sensitive to *S. glutinosa* and *S. transsylvanica* and *A. fumigatus* to *S. glutinosa*. Based on MIC values, the antifungal potency was *S. officinalis* > *S. glutinosa* > *S. transsylvanica*. As an exception, the toxigenic strain *A. ochraceus* was more sensible to *S. transsylvanica* than *S. glutinosa* (MIC 0.45 mg mL^−1^ vs 0.75 mg mL^−1^). *S. glutinosa* was the most effective on *T. viride* (MIC 0.37 mg mL^−1^) a well-known fungus used as a biofertilizer, as an agent for pathogens biocontrol and as a plant growth promoter. The minimal fungicidal concentration was directly related to the MIC sensitivity and results twofold the MIC concentration for all tested extracts against all tested strains.

The effects of antifungal activity of Salvia species could be marginally compared to literature data that confirm this as an unexplored field for experimental research. Few scientific studies investigated the ability of the essential oil of *S. officinalis* to inhibit the fungal growth, against the toxigenic *Fusarium* sp., several strains related to the crop production and food storage (*Botritis cinerea*, *Verticillium dahliae* and *Penicillium aurantiogriseum*), against *Candida* and other dermatophyte strains [45,46,47,48,49,50]. Moreover, Veličković et al. [16] evaluated the antifungal potential of methanolic and ethanolic extracts obtained from *S. glutinosa* and *S. officinalis* aerial parts using the agar-well diffusion method. The highest activity was against the yeasts (*S. cerevisiae*, *C. albicans*), while there was no activity against the mold *A. niger*.

Concerning antimicrobial activity of *S. transsylvanica*, our study reports for first time data about antibacterial, antiquorum and antifungal potential of the extracts obtained from these species. The obtained results are a preliminary basis for further investigations that could offer evidence-based information about the use of *S. transsylvanica* as a potential source of antibacterial agents with applications in human health, food preservation and food industry.

### 3.5. Cytotoxic Effects of Salvia Extracts on Cell Lines

**Figure 1:**
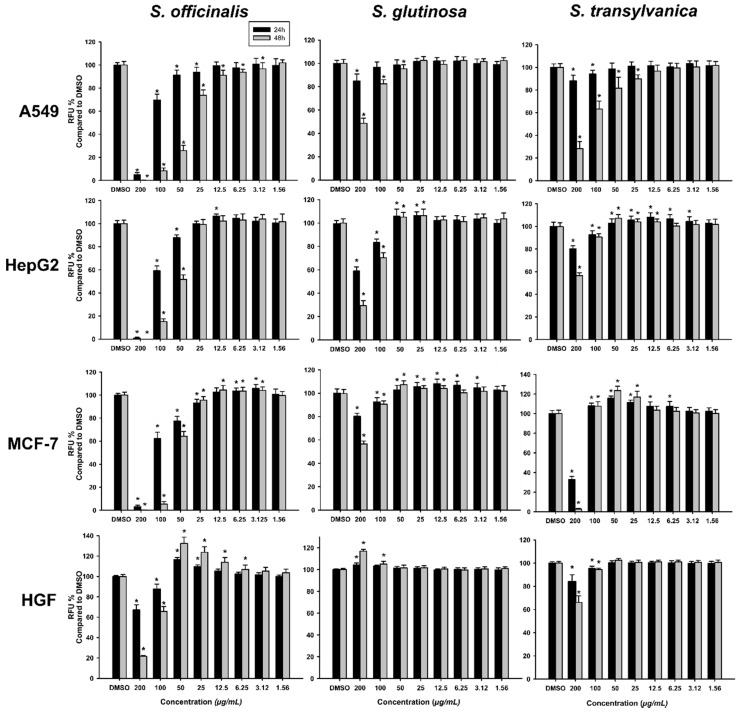
Cytotoxic effect of the *Salvia* sp. extracts observed using alamarBlue assay on the cancerous cell lines A549, HepG2, MCF-7 and on normal cells HGF. The results are expressed as relative means ± standard deviations (6 technical replicates for each of the 3 biologic replicates) where the negative control (DMSO 0.2%) is 100%. (*) indicates significant differences compared to negative control (ANOVA + Dunnett’s; *p* < 0.05).

Exposure of the cancerous cell lines to the *Salvia* extracts resulted in a decrease of the cellular viability dependent on the concentration, time of exposure and the type of *Salvia* extract used. From the three extracts, the *S. officinalis* extract was the most active, statistically decreasing the viability at 24 h from 25 μg/mL on A549 and MCF-7 and from 50 μg/mL on HepG2. At intermediary doses (25–6.25 μg/mL) no major differences were observed between 24 h and 48 h post-exposure to *S. officinalis* extract, but at higher doses, a drastic increase in cytotoxicity was observed at 48 h post-exposure in all cancerous cell lines. The other two *Salvia* extracts behaved similarly, displaying a modest cytotoxic effect, present at the highest doses used. Interestingly, an increase in the viability was observed for *S. glutinosa* and *S. transsylvanica* at intermediary doses on the HepG2 and MCF-7 cells. This increased viability could be the result of a hormetic effect, non-toxic doses of *Salvia* extracts increased the metabolism of the cells, this biologic function being assayed using Alamar Blue assay (Figure 1). The calculated IC_50_ values are presented in Table 9.

Similar results, with close IC_50_, were reported by Jiang et al. [51], who evaluated the effects of *S. officinalis* and *S. miltiorrhiza* extracts on HepG2 cells and normal human liver cells. In comparison with the cancerous cell lines, the normal human gingival fibroblasts (HGF) were more resilient to the cytotoxic potential of the *Salvia* sp. extracts. From the three extracts tested, the *S. officinalis* extract presented the highest toxicity towards the HGF cells, followed by the *S. transsylvanica* and *S. glutinosa* extracts (Table 9). At intermediary doses, in a similar manner to the one observed on cancerous cell lines, exposure to the *Salvia* extracts induced a hormetic response (Figure 1).

## 4. Conclusions

A large number of *Salvia* species have been used for centuries as important sources of natural compounds with applications in health, cosmetic and dietary purposes. Among this species, *S. officinalis* is the most known and studied, being rich in volatile oil, phenolic and terpenoidic compounds responsible for several bioactivities (i.e., antibacterial, antioxidant, anti-inflammatory, neuroprotective) proven by in vitro and in vivo studies. In the last decades, the phytochemical profile and potential bioactivities of some less-known *Salvia* species have been investigated, certifying their health-related benefits.

In this regard, our present study made a comparative analysis between the phytochemical profile and potential bioactivities of well-established *S. officinalis* and less-studied *S. glutinosa* and *S. transsylvanica*. HPLC analyses revealed the presence of high amounts of catechin and epicatechin in *S. officinalis* extract, while rutin was found as the main compound of *S. glutinosa* and *S. transsylvanica*. The antioxidant capacity of the extracts was correlated with high TPC and TFC values, highlighting the contribution of phenolic and flavonoidic compounds to the global antioxidant activity of this species. In vitro enzyme inhibition assays emphasized the neuroprotective and antidiabetic potential of *S. officinalis* (the most active as AChE, BChE and *α*-glucosidase inhibitor), *S. glutinosa* (the most active as *α*-amylase inhibitor) and *S. transsylvanica*. The highest antibacterial and antifungal activity was exerted by *S. officinalis*, followed by *S. glutinosa* and *S. transsylvanica*, a similar trend being observed for the cytotoxic activity as well.

Based on comprehensive research of the literature, we can affirm that our research contributes to the actual knowledge about the chemical composition and potential bioactivities of *S. officinalis*; moreover, we reported for the first time the in vitro neuroprotective, antidiabetic and cytotoxic potential of *S. glutinosa* and *S. transsylvanica* extracts. The obtained results are a promising starting point for future deep investigations that could offer new perspectives for the use of this species as valuable sources of natural compounds with applications in the field of nutraceuticals and dietary supplements.

## Figures and Tables

**Table 1:** Phenolic compounds identified and quantified in *S. transsylvanica, S. glutinosa* and *S. officinalis* extracts (µg/g dry extract).

	Compound	*S. transsylvanica*	*S. glutinosa*	*S. officinalis*
1	Catechin	1911.1 ± 11.2	1292.1 ± 207.4	1112.6 ± 201.7
2	Chlorogenic acid	149.5 ± 19	106.3 ± 32.1	NF
3	*p*-OH benzoic acid	113.9 ± 21.7	182.3 ± 36.9	89.1 ± 1.7
4	Epicatechin	569.2 ± 25	NF	1659.1 ± 10.8
5	*p*-Coumaric acid	284.3 ± 40.8	998.9 ± 239.5	NF
6	Rutin	3034.9 ± 31.8	4070.2 ± 636.5	1357.9 ± 34.4
7	Naringin	304.9 ± 13.1	668.9 ± 55.9	222.1 ± 22.6
8	Quercetin	156.6 ± 11.3	130.5 ± 13.7	332.5 ± 20.3
9	Naringenin	NF	828.5 ± 253.9	NF
10	Carvacrol	239.3 ± 50.7	183.3 ± 0.3	164.3 ± 1.5
	Total (μg/g)	6763.7 ± 224.6	8461 ± 1476.2	4937.6 ± 293

Note: results are presented as mean ± SD (standard deviation); all samples were analyzed in triplicate. NF: not found.

**Table 2:** Total phenolic content (TPC) and total flavonoidic content (TFC) of *S. transsylvanica, S. glutinosa* and *S. officinalis* aerial parts extracts.

Samples	TPC (mg GAE/g Extract)	TFC (mg RE/g Extract)
*S. glutinosa*	37.74 ± 0.69	16.03 ± 0.40
*S. officinalis*	65.02 ± 2.44	31.80 ± 0.24
*S. transsylvanica*	20.32 ± 1.01	13.94 ± 0.55

Note: results are presented as mean ± SD (standard deviation); all samples were analyzed in triplicate.

**Table 3:** In vitro antioxidant capacity of the extracts obtained from *S. transsylvanica, S. glutinosa* and *S. officinalis*.

Samples	Phosphomolybdenum Assay (mmolTE/g Extract)	DPPH (mgTE/g Extract)	ABTS (mgTE/g Extract)	CUPRAC (mgTE/g Extract)	FRAP (mgTE/g Extract)	Metal Chelating Activity (mgEDTAE/g Extract)
*S. glutinosa*	1.58 ± 0.14	80.42 ± 0.95	126.70 ± 5.22	175.91 ± 6.18	130.59 ± 4.71	9.92 ± 0.44
*S. officinalis*	2.99 ± 0.46	194.85 ± 0.40	358.56 ± 6.37	400.01 ± 4.41	329.32 ± 8.04	6.22 ± 0.96
*S. transsylvanica*	0.90 ± 0.02	59.29 ± 2.37	77.53 ± 2.25	118.11 ± 4.17	90.94 ± 2.55	6.18 ± 0.56

Note: results are presented as mean ± SD (standard deviation); all samples were analyzed in triplicate.

**Table 4:** Enzyme inhibitory potential of the extracts obtained from *S. transsylvanica, S. glutinosa* and *S. officinalis* aerial parts extracts.

Samples	AChE Inhibition (mgGALAE/g Extract)	BChE Inhibition (mgGALAE/g Extract)	*α*-Amylase Inhibition (mmolACAE/g Extract)	*α*-Glucosidase Inhibition (mmolACAE/g Extract)	Tyrosinase Inhibition (mgKAE/g Extract)
*S. glutinosa*	1.70 ± 0.16	0.52 ± 0.10	0.74 ± 0.01	21.54 ± 1.29	NA
*S. officinalis*	1.97 ± 0.06	2.40 ± 0.16	0.63 ± 0.05	27.01 ± 0.12	NA
*S. transsylvanica*	1.72 ± 0.09	1.43 ± 0.19	0.65 ± 0.01	25.62 ± 1.10	NA

Note: results are presented as mean ± SD (standard deviation); all samples were analyzed in triplicate. AChE = Acetylcholinesterase; BChE = butyrylcholinesterase; GALAE = galantamine equivalents; ACAE = acarbose equivalents; KAE = kojic acid equivalents; NA = no activity.

**Table 5:** Antibacterial activity of the extracts obtained from *S. transsylvanica, S. glutinosa* and *S. officinalis* (mg/mL).

		Gram-Negative Bacteria				Gram-Positive Bacteria			
		*E. coli*	*P. aeruginosa*	*S. typhimurium*	*L. monocytogenes*	*E. cloacae*	*M. flavus*	*B. cereus*	*S. aureus*
*S. officinalis*	MIC	0.045	0.09	0.09	0.18	0.01	0.18	0.18	0.18
	MBC	0.09	0.18	0.18	0.36	0.02	0.36	0.36	0.36
*S. transsylvanica*	MIC	1.1	1.5	0.75	1.5	1.5	0.75	0.75	0.75
	MBC	2.2	3	1.5	3	3	1.5	1.5	1.5
*S. glutinosa*	MIC	0.6	1.5	0.75	1.5	0.38	0.75	0.09	1.5
	MBC	1.2	3	1.5	3	0.75	1.5	0.18	3
Ampicillin	MIC	0.18	0.4	0.13	0.2	0.17	0.13	0.17	0.1
	MBC	0.27	0.67	0.2	0.33	0.2	0.15	0.2	0.2
Streptomycin	MIC	0.13	0.13	0.17	0.22	0.03	0.07	0.03	0.13
	MBC	0.2	0.23	0.27	0.4	0.07	0.17	0.07	0.3

Note: MBC = minimum bactericidal concentration; MIC = minimum inhibitory concentration.

**Table 6:** Percentage of inhibition of biofilm formation after the treatment with subinhibitory concentrations of the extracts obtained from *S. transsylvanica, S. glutinosa* and *S. officinalis*.

Sample	Inhibition Rate		
	1/2 MIC	1/4 MIC	1/8 MIC
*S. officinalis*	61.1	27.5	13.9
*S. transsylvanica*	48.4	34.9	NE
*S. glutinosa*	30.5	19.6	7.8
Ampicillin	43.5	30.9	7.8
Streptomycin	50.7	29	11.4

Note: results are expressed as percentage; NE = no effect.

**Table 7:** Reduction of pyocyanin pigment production in *P. aeruginosa* PAO1 incubated in medium supplemented with 0.5-MIC concentrations of the extracts.

Sample	Inhibition Rate (%)
*S. officinalis* (0.9 mg/mL)	NE
*S. transsylvanica* (0.75 mg/mL)	28
*S. glutinosa* (0.75 mg/mL)	NE
Ampicillin (0.2 mg/mL)	19
Streptomycin (0.65 mg/mL)	21

Note: results are expressed as percentage; NE = no effect.

**Table 8:** Antifungal activity of the extracts obtained from *S. transsylvanica, S. glutinosa* and *S. officinalis* (mg/mL).

Samples		*A. fumigatus*	*A. versicolor*	*A. ochraceus*	*A. niger*	*T. viride*	*P. funiculosum*	*P. ochrochlorum*	*P. verrucosum*
*S. officinalis*	MIC	0.4	0.2	0.4	0.4	0.4	0.06	0.4	0.12
	MFC	0.8	0.4	0.8	0.8	0.8	0.12	0.8	0.24
*S. transsylvanica*	MIC	0.9	0.9	0.45	0.9	0.45	0.9	1.8	0.9
	MFC	1.8	1.8	0.9	1.8	0.9	1.8	3.6	1.8
*S. glutinosa*	MIC	1.5	0.75	0.75	0.75	0.37	0.75	3	0.75
	MFC	3	1.5	1.5	1.5	0.75	1.5	6	1.5
Ketoconazole	MIC	0.2	0.2	0.15	0.2	1	0.2	1	0.2
	MFC	0.5	0.5	0.2	0.5	1.5	0.5	1.5	0.3
Bifonazole	MIC	0.15	0.1	0.15	0.15	0.15	0.2	0.2	0.1
	MFC	0.2	0.2	0.2	0.2	0.2	0.25	0.25	0.2

Note: MFC = minimum fungicidal concentration; MIC = minimum inhibitory concentration.

**Table 9:** IC_50_ values (µg extract/mL) after exposure of human lung adenocarcinoma (A549), human hepatocellular carcinoma (HepG2), human breast cancer (MCF-7) and human normal gingival fibroblasts (HGF) to the extracts of *S. transsylvanica, S. glutinosa* and *S. officinalis* for 24 h and 48 h.

Samples	IC_50_ (µg/mL)							
	24 h				48 h			
	HepG2	A549	MCF-7	HGF	HepG2	A549	MCF-7	HGF
*S. glutinosa*	>200	>200	>200	>200	100.2 ± 3.4	>200	>200	>200
*S. officinalis*	177.9 ± 66.1	118.7 ± 37.5	>100	>200	50.9 ± 2.4	35.5 ± 1.9	56.4 ± 2.0	110.7 ± 7.7
*S. transsylvanica*	>200	>200	>100	>200	>200	163.3 ± 23.2	>100	>200
